# Copy Number Variations in a Cohort of 420 Individuals with Neurodevelopmental Disorders From the South of Brazil

**DOI:** 10.1038/s41598-019-54347-z

**Published:** 2019-11-28

**Authors:** Tiago Fernando Chaves, Nathacha Baretto, Luan Freitas de Oliveira, Maristela Ocampos, Ingrid Tremel Barbato, Mayara Anselmi, Gisele Rozone De Luca, Jorge Humberto Barbato Filho, Louise Lapagesse de Camargo Pinto, Pricila Bernardi, Angelica Francesca Maris

**Affiliations:** 1 Universidade Federal de Santa Catarina, Florianópolis, SC Brazil; 2 Laboratory Neurogene, Florianópolis, SC Brazil; 3 Children’s Hospital Joana de Gusmão, Florianópolis, SC Brazil; 4 University Hospital Professor Polydoro Ernani de São Thiago, Florianópolis, SC Brazil

**Keywords:** Genetic testing, Cytogenetics, Genetic counselling, Autism spectrum disorders

## Abstract

Chromosomal microarray (CMA) is now recommended as first tier for the evaluation in individuals with unexplained neurodevelopmental disorders (ND). However, in developing countries such as Brazil, classical cytogenetic tests are still the most used in clinical practice, as reflected by the scarcity of publications of microarray investigation in larger cohorts. This is a retrospective study which analyses the reading files of CMA and available clinical data from 420 patients from the south of Brazil, mostly children, with neurodevelopmental disorders requested by medical geneticists and neurologists for diagnostic purpose. Previous karyotyping was reported for 138 and includes 17 with abnormal results. The platforms used for CMA were CYTOSCAN 750K (75%) and CYTOSCAN HD (25%). The sex ratio of the patients was 1.625 males :1 female and the mean age was 9.5 years. A total of 96 pathogenic copy number variations (CNVs), 58 deletions and 38 duplications, were found in 18% of the patients and in all chromosomes, except chromosome 11. For 12% of the patients only variants of uncertain clinical significance were found. No clinically relevant CNV was found in 70%. The main referrals for chromosomal microarrays (CMA) were developmental delay (DD), intellectual disability (ID), facial dysmorphism and autism spectrum disorder (ASD). DD/ID were present in 80%, facial dysmorphism in 52% and ASD in 32%. Some phenotypes in this population could be predictive of a higher probability to carry a pathogenic CNV, as follows: dysmorphic facial features (*p*-value = < 0.0001, OR = 0.32), obesity (*p*-value = 0.006, OR = 0.20), short stature (*p*-value = 0.032, OR = 0.44), genitourinary anomalies (*p*-value = 0.032, OR = 0.63) and ASD (*p*-value = 0.039, OR = 1.94). The diagnostic rate for CMA in this study was 18%. We present the largest report of CMA data in a cohort with ND in Brazil. We characterize the rare CNVs found together with the main phenotypes presented by each patient, list phenotypes which could predict a higher diagnostic probability by CMA in patients with a neurodevelopmental disorder and show how CMA and classical karyotyping results are complementary.

## Introduction

Neurodevelopmental disorders (ND), which mostly involve developmental delay (DD), intellectual disability (ID) and/or autism spectrum disorders (ASD), affect around 3–4% of the world’s population^1,2^. Such disorders, when isolated, are termed non-syndromic; when associated with the presence of dysmorphisms or apparent congenital anomalies (CA), are termed syndromic^3^.

Individuals affected with ND usually present reduced adaptive skills and/or limited intellectual ability and face major challenges throughout their life, often including motor difficulties, CA and problems with social interaction. These are relevant characteristics which affect not only the patient, but also impact the daily life of family members due to their special care and dedication needs^3,4^.

Adequate diagnosis is necessary for the clinical follow-up of individuals with ND and to provide appropriate genetic counseling to the family, preventing the risk of recurrence. Hundreds of genes and many different chromosomal changes are associated with ND and, apart from the well-known and easy identifiable syndromes, the diagnosis of each affected individual remains a clinical challenge.

Due to their high phenotypic and genetic heterogeneity, studies and diagnostics of ND are intricate. Additionally both, genetic and environmental factors, isolated or together, play an important role in their pathogenesis^5,6^. Currently, molecular karyotyping by chromosomal microarrays (CMA) has been clinically recommended as the first-tier cytogenetic diagnostic test of choice in the investigation of patients with idiopathic ND, such as developmental delay, intellectual disability, autism spectrum disorder and multiple congenital anomalies^7^.

After the publication of the first comprehensive map of copy number variation in the human genome^8^, that lead the authors to suggest that CNV assessment should become standard in the design of all studies of the genetic basis of phenotypic variation, including disease susceptibility, a growing number of publications have reported the diagnostic yield of CMA in cohorts of patients with ND, with a worldwide average rate of 15% to 20% in recent years^5,9–18^, (Table 1).

**Table 1:** Some recent studies that used chromosomal microarrays for diagnostic testing in cohorts of affected individuals and their diagnostic rates.

Study/Year	Cohort	CMA Platforms	Sample No	Detection Rate of Pathogenic CNVs
Bruno *et al*.^53^	Australia patents with ID and CA	AFFYMETRIX 250K microarrays	117	15%
Kashevarova *et al*.^11^	Russian patients with ID	AGILENT 44K e 60K.	79	28%
Bartnik *et al*.^13^	Polish patients with neurodevelopmental disorder	V8 OLIGO 180k (customized)	256	16%
Preiksaitiene *et al*.^15^	Lithuanian patients with neurodevelopmental disorder	AGILENT 105k and 400k	201	14%
Roselló *et al*.^5^	Spanish children with neurodevelopmental disorder	AGILENT 44K	246	30%
Coutton *et al*.^14^	French children with moderate ID	4 × 180K OLIGONUCLEOTIDE ARRAY (AGILENT TECHNOLOGIES)	66	21%
Lay-Son *et al*.^16^	Chilean patients with neurodevelopmental disorder	CYTOSCAN HD, AFFYMETRIX	40	25%
Pfundt *et al*.^9^	North American patients with disorder neurodevelopment	CYTOSCAN DX, AFFYMETRIX (Platform similar to CYTOSCAN HD)	960	14% (first line test)*
Quintela *et al*.^26^	Galician patients with neurodevelopmental disorder (Spain)	CYTOGENETICS WHOLE-GENOME 2.7 M (n = 126) and CYTOSCAN HD (n = 447)	573	11,2% to 13,6%
Wu *et al*.^12^	Children with congenital heart disease (Chinese population)	AFFYMETRIX CYTOSCAN HD	104	28%
Borlot *et al*.^24^	Patients with unexplained childhood-onset epilepsy and intellectual disability (Toronto)	4 × 180K OLIGONUCLEOTIDE ARRAY (AGILENT TECHNOLOGIES)	134	16%
HEIDE *et al*.^27^	Patients with both corpus callosum abnormality and intellectual disability (French)	370 CNV-QUAD (n = 7), CYTOSNP-12 (n = 121) our HUMANOMNIEXPRESS-24 (n = 21) (ILLUMINA)	149	13%
Di Gregorio *et al*.^54^	Patients diagnosed with DD/ID in Turin, Italy	AGILENT 60K	1015	11%
Sansović *et al*.^55^	Patients with DD/ID with or without dysmorphism, ASD, and/or CA (Croatia)	AGILENT SUREPRINT G3 UNRESTRICTED CGH ISCA V2	337	22%
Kessi *et al*.^56^	Patients with ID/DD and epilepsy (Chinese population)	AFFYMETRIX + SNP Microarray And ILLUMINA HUMANCYTOSNP-12	100	25%

^*^CMA used as a first line test (no screening with classical cytogenetics). CYTOSCAN HD platform validation study at FDA. DD = developmental delay; ID = intellectual disability; CA = congenital anomalies; ASD = autism spectrum disorder.

Although the CMA test is considered the gold standard in the diagnostics of ND, in Latin America classic karyotyping is still the predominant genetic test in clinical practice, and in Brazil there are only a few publications of CMA in cohorts of ND patients. Pereira and coworkers^7^ analyzed 15 patients with ND attended by the Laboratory of Human Cytogenetics and Molecular Genetics of the PUC (Pontifical Catholic University) of Goiás between 2010 and 2012, with a diagnostic rate of 22% using the CYTOSCAN HD platform. In Espírito Santo, Pratte-Santos and coworkers^19^ investigated 39 individuals with ND and a normal karyotype, with the 4 × 180K CMA platform from Agilent, reporting a 15% rate of pathogenic CNVs. In the Northeast of Brazil, Vianna and coworkers^20^, using a 60K microarray platform (Agilent) in 200 patients with ND, found pathogenic CNVs in 33 of them, a diagnostic rate of 16.5%.

Our study analyzed a cohort of 420 patients from the south of Brazil, that underwent microarray testing from 2013–2016 for diagnostic purpose.

## Results

Of the 420 participating patients, 260 (62%) were male and 160 (38%) female, from 0 to 49 years of age, with a mean age of 9.5 years (SD = 9.73, Mo = 4). For 139 patients previous karyotyping was reported, 122 with normal result and 17 with abnormal results for which CMA was requested to define the sequences involved.

For most patients’ previous genetic assessments are unclear.

From the 420 microarrays, a total of 2,468 CNVs which fulfilled the filtering criteria were selected; 1,462 duplications and 1,007 deletions which were interpreted and classified into benign CNVs, pathogenic CNVs and variants of uncertain clinical significance (VOUS).

**Figure 1:**
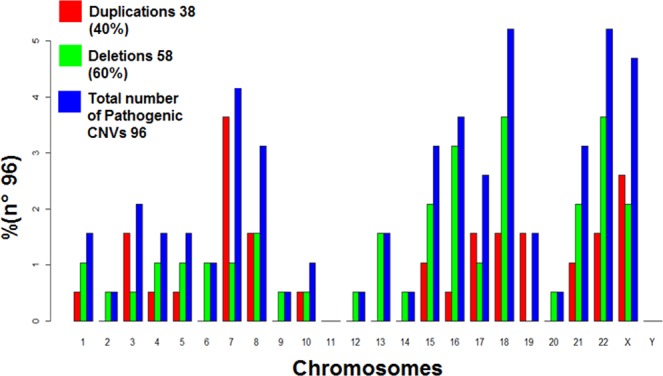
Pathogenic CNVs per chromosome.

In 18% patients (75/420) we identified a total of 96 rare CNVs which were interpreted as pathogenic (Table 2). Of these 75 patients, 15 had more than one pathogenic CNV, 9 of them had 2 pathogenic CNVs (#33, #47, #61, #127, #251, #332, #372 and #407) and 6 had 3 pathogenic CNVs (#151, #188, #196, #219, #270 and #392). Three cases (#81, #255 and #331), along with a pathogenic CNV, also presented VOUS. Of the 96 pathogenic CNVs 58 were deletions, leaving only a single copy of the sequence involved. The remaining 38 were duplications that usually result in a total of three copies of the sequence involved, however in two brothers (cases #24 and #25) the duplication of a relevant region of chromosome X resulted in two copies (in which the main reason of pathogenicity is the fact that none of the duplicated copies undergoes X-inactivation, as usual in females) and in three patients (cases #306, #422 and #443) the CNV found was in a four-copy state, of which case #422 had a previous abnormal karyotype result (Table 2). The pathogenic CNVs were found in all chromosomes, except in chromosome 11. Figure 1 illustrates the frequency and number of pathogenic CNVs found per chromosome.

**Table 2:** Pathogenic CNVs found in the cohort.

Case	CNV	Microarray Nomenclature	Size (Kbp)	N° of Genes	Some of the Relevant Genes	Phenotype	Gender/other info	Inheritance	Karyotype	Syndrome
#9	Del	arr[hg19] 13q33.1q34(104,782,510–112,352,804)x1	7.570	26	COL4A2, DAOA-	DD, LDO, FD, low weight, microcephaly and mot dif	M/−	ND		—
#15	Del	arr[hg19] 16p11.2(28,689,085–29,043,863)x1	355	18	SH2B1	DD, Aut	M/Affected brother (#16)	ND		distal 16p11.2 deletion syndrome
#16	Del	arr[hg19] 16p11.2(28,689,085–29,388,495)x1	362	18	SH2B1	DD, Aut	M/Affected brother (#15)	ND		distal 16p11.2 deletion syndrome
#18	Del	arr[hg19] 6q15-q21(93,082,925–110,504,681)x1	17.422	66	SIM1, SEC. 63	DD, CA, FD, SLD	M/Affected brother (#19 in Table 2)	ND		—
#24	Dup	arr[hg19] Xq27.3q28(146,425,635–151,604,987)x2	5.179	40	FMR1, AFF2	DD, SLD, FD and obesity	M/Affected brother (#25)	ND		—
#25	Dup	arr[hg19] Xq27.3q28(146,418,810–151,604,987)x2	5.186	40	FMR1, AFF2	DD, SLD, FD and obesity	M/Affected brother (#24)	ND		—
#26	Del	arr[hg19] 22q11.21(18,648,855–21,269,224)x1	2.620	58	TBX1	DIL, mot dif, hyperactivity	F/−	ND		Di George syndrome
#33	Del	arr[hg19] 18p11.32p11.23(136,227–8,348,006)x1	8.212	43	TGIF	SID, mot dif and FD and hypotonia	F/*2Pv	ND		18p deletion syndrome
#33	Del	arr[hg19] 18q22.2q23(67,357,305–78,013,728)x1	10.656	43	RTTN, CTDP1	SID, mot dif and FD and hypotonia	F/*2Pv	ND		—
#44	Del	arr[hg19] 22q13.2q13.33(43,600,479–51,197,766)x1	7.597	95	UPK3A, FBLN1, SHANK3	DIM, Mot Dif, FD and CA	F/−	ND	46, XX, del(22)(q13)	Phelan-McDermid Syndrome
#47	Del	arr[hg19] Xp22.33(372,029–578,764)x1	207	0	SHOX	Short stature	F/*2Pv	ND		Leri-Weill dyschondrosthosis syndrome
#47	Del	arr[hg19] Xp22.33(679,520-950,907)x1	271	0	SHOX	Short stature	F/*2Pv	ND		Leri-Weill dyschondrosthosis syndrome
#52	Del	arr[hg19] 22q13.33(50,788,193–51,115,526)x1	327	18	SHANK3	SID, Aut, mot dif, FD, CA and epilepsy.	M/	ND		Phelan-McDermid syndrome
#56	Del	arr[hg19] 8p21.1p11.21(28,393,484–41,026,001)x1	12.632	69	NRG1, FGFR1, ANK1	DIL, FD	M/−	ND	XY, 46, del(8)(p21-p11)	8p intersticial deletion including p12 syndrome
#61	Del	arr[hg19] 13q34(114,141,294–115,107,733)x1	966	15	TFD1, GRK1, RASA3, GAS6, CHAMP1	DIL, hyperactivity	M/*2Pv/4 affected siblings	ND		Distal 13q deletion
#61	Dup	arr[hg19] 8p23.3p23.1(158,048–8,142,435)x3	7.984	64	ARHGEF10, MCPH1	DIL, hyperactivity	M/*2Pv/4 affected siblings	ND		Distal trisomy 8p
#66	Dup	arr[hg19] 15q25.1q26.3(80,304,866–102,429,040)x3	22.124	175	AKAP13, CPEB1, NTRK3, WDR73	SID, Aut, convulsions, SLD, hyperactivity, one kidney and FD	M/−	ND		—
#69	Del	arr[hg19] 16p12.2p11.2(21,405,327–29,388,495)x1	7.983	82	SH2B1	DIL, Aut, SLD, hyperactivity and FD	M/−	ND		—
#70	Dup	arr[hg19] 7q11.23(72,732,834–74,155,067)x3	1.422	27	WBSCR27, WBSCR28	DIM, Aut and hyperactivity	M/−	ND		—
#76	Dup	arr[hg19] 7q11.23(72,556,215–74,245,599)x3	1.689	34	WBSCR27, WBSCR28	DIL, Aut	M/−	ND		Williams-Beuren region duplication syndrome
#77	Del	arr[hg19] 15q13.2q13.3(31,073,735–32,446,830)x1	1.373	9	CHNA7	DIL, Aut and hyperactivity	M/−	ND		—
#81	Del	arr[hg19] 17q21.31(43,574,907–44,212,415)x1	637	11	KANSL1	SLD, convulsions and FD	M/*VOUS	ND		—
#85	Dup	arr[hg19] 7q31.32q33(122,739,692–136,150,625)x3	1.341	101	WASL	DIL, mot dif, hyperactivity, FD and CA	F/−	ND		—
#91	Dup	arr[hg19] 16p13.3p13.12(85,880–14,524,038)x3	14.438	262	CREBBP	SID, Aut	M/−	ND		16p13.3 microduplication syndrome
#93	Del	arr[hg19] 3p14.1p13(68,988,297–70,938,968)x1	1.951	8	MITF, TMF1	Deafness, ophthalmopathy and ADHD	F/−	ND		Waardenburg syndrome type II
#95	Del	arr[hg19] 22q11.21(18,648,855-21,058,888)x1	2.410	55	TBX1	DIL, mot dif and hyperactivity	F/	ND		Di George syndrome
#102	Del	arr[hg19] Xp22.31(6,449,752–8,135,644)x1	1.715	7	STS	DD and FD	F/−			—
#105	Del	arr[hg19] 1p36.33p36.32(1,073,574-2,458,606)x1	1.385	54	GABRD, SKI	DIM and FD	M/−			—
#107	Dup	arr[hg19] 7p21–p22.3 (43,376–9,454,786)x3	9.411	145	RNF216	MID, convulsions and FD	M/			—
#113	Del	arr[hg19] 16p13.3(85,880–2,145,951)x1	2.060	108	TSC2, SOX8	DD, FD and tuberous sclerosis	F/affected twin sister (#115)	ND		ATR-16 syndrome (#141750) (thalassemia/mental retardation syndrome)
#115	Del	arr[hg19] 16p13.3(85,880–2,146,448)x1	2.060	108	TSC2, SOX8	DD, FD and tuberous sclerosis	F/affected twin sister (#113)	ND		ATR-16 syndrome – (#141750) (thalassemia/mental retardation syndrome)
#116	Dup	arr[hg19] Xq26.3q28(135,224,845–155,233,098)x2	20.008	212	GPR101, fmr1, fmr2, MECP2, RAB39B, FLNA, GDI1	Low weight, abnormal growth, prematurity, CA, DD, FD and microcephaly	M/−	ND	46, XY, add(22q)	Xq26.3, Xq27.3-q28 and Xq28 duplication syndromes
#127	Dup	arr[hg19] 10q25.1q26.3(108,553,165–135,427,143)x3	26.873	182	101 OMIMs	Low weight, CA, DD, ID, epilepsy and FD	F/*2Pv	46, XX, der(18)t(10; 18) (q25.2; q22,2) mat	46, XX, add(18)(q23)	distal trisomy 10q syndrome
#127	Del	arr[hg19] 18q22.3q23(69,055,745–78,014,123)x1	8.958	36	62 OMIMs	Low weight, CA, DD, ID, epilepsy and FD	F/*2Pv	46, XX, der(18)t(10; 18) (q25.2; q22,2) mat.	46, XX, add(18)(q23)	18q deletion syndrome
#130	Del	arr[hg19] 15q11.2(22,770,421–23,209,654)x1	732	6	NIPA1, NIPA2, CYF1P1, TUBGCP5	Short stature, DD, ADHD and FD	M/−	ND		15q11.2 BP1–BP2 microdeletion syndrome (OMIM 615656)
#148	Del	arr[hg19] Xp22.3q28(1–247,249,719)x3 ou arr(X)x3	155.270	—	—	DD, Aut and schizophrenia	F/−	ND		Triple X syndrome
#149	Del	arr[hg19] 5p15.31p14.3(6,801,589–18,992,827)x1	12.191	40	—	Hypotonia, DD, SLD, behavioral disorder and FD	F/Sister of #445	46, XY, t(1; 2)(q44;~p23-pter); t(5; 7)(p14.3-p15.31; p22) pat.		Cri du-Chat syndrome
#151	Del	arr[hg19] 18p11.32p11.31(136,226–4,409,550)x1	4.273	26	TGIF1, SMCHD1	Short stature, FD, IUGR and DD	M/*3Pv	ND		—
#151	Dup	arr[hg19] 7p22.3p21.3(43,376–9,454,786)x3	9.411	92	50 OMIMs	Short stature, FD, IUGR and DD	M/*3Pv	ND		—
#151	Dup	arr[hg19] Xq28(151,356,116–155,233,731)x2	3.877	106	MECP2, L1CAM	Short stature, FD, IUGR and DD	M/*3Pv	ND		Xq28 duplication syndrome
#160	Del	arr[hg19] 2q31.1-q31.2(174,065,715–190,659,870)x1	16.582	160	HOXD, CHN1	Short stature, DD, ID, SLD, epilepsy and FD	F/−	ND		2q31.1 microdeletion syndrome
#169	Del	arr[hg19] 18p11.32p11.21(136,226–15,181,666)x1	15.045	86	TGIF1, SMCHD1	ID, FD and short stature	F/−	De Novo		partial 18p deletion syndrome
#171	Dup	arr[hg19] 7q31.32q33(122,739,692–136,150,625)x3	13.411	101	LEP	DD and ID	F/−	ND		partial trisomy 7q31.32q33
#181	Del	arr[hg19] 22q13.31q13.33(46,168,628–51,115,526)x1	4.947	66	SHANK3	Slender build, hypotonia, convulsions, DD and FD	F/−	ND		Phelan-McDermid syndrome
#184	Del	arr[hg19] 15q11.2q13.1(22,770,421–28,823,722)x1	6.053	121	UBE3A, SNRPN	DD, ID, epilepsy, Aut and ADHD	M/−	ND		Angelman/Prader - Willi syndrome
#188	Dup	arr[hg19] 19p13.3(1,712,849–6,074,347)x3	4.361	131	SEMA6B, MAP2K2	DD, FD and microcephaly	M/*3Pv	ND		partial trisomy 19p13
#188	Dup	arr[hg19] 19p13.3(260,911–1,434,508)x3	1.174	52	—	DD, FD and microcephaly	M/*3Pv	ND		partial trisomy 19p13
#188	Del	arr[hg19] 21q22.3(46,597,460–48,097,372)x1	1.450	24	—	DD, FD and Microcephaly	M/*3Pv	ND		—
#196	Dup	arr[hg19] 18q21.2q22.1(49,094,563–66,586,144)x3	17.492	68	PIGN	Short stature, CAs, DD, SLD and FD	F/*3Pv	ND	46, XX, 5p+	partial trisomy 18q
#196	Dup	arr[hg19] 18q22.1q23(66,593,317–78,014,123)x3	11.421	41	PIGN	Short stature, CAs, DD, SLD and FD	F/*3Pv	ND	46, XX, 5p+	Distal trisomy 18q
#196	Del	arr[hg19] 5p15.33p15.2(113,576–12,747,875)x1	12.634	72	CTNND2, TERT	Short stature, CAs, DD, SLD and FD	F/*3Pv	ND	46, XX, 5p+	Cri du-Chat syndrome
#216	Del	arr[hg19] 17q21.31(43,703,801–44,212,416)x1	508	9	KANSL1	Low weight, short stature, fanconi anemia, DD, SLD and FD	F/−	ND		Koolen de Vries syndrome
#219	Dup	arr[hg19] 8p11.22p11.21(39,388,765–42,335,424)x3	2.946	22	18 OMIMs	Short stature, prematurity, IUGR, DD and FD	F/*3Pv	ND	46, XX, add(8)(p23.1)	8p inverted duplication/deletion [invdupdel(8p)] syndrome
#219	Dup	arr[hg19] 8p23.1p11.22(11,935,023–39,246,760)x3	27.311	191	119 OMIIMs	Short stature, prematurity, IUGR, DD and FD	F/*3Pv	ND	46, XX, add(8)(p23.1)	8p inverted duplication/deletion [invdupdel(8p)] syndrome
#219	Del	arr[hg19] 8p23.3p23.1(158,048–6,940,661)x1	6.782	31	15 OMIMs	Short stature, prematurity, IUGR, DD and FD	F/*3Pv	ND	46, XX, add(8)(p23.1)	8p inverted duplication/deletion [invdupdel(8p)] syndrome
#235	Dup	arr[hg19] 17p11.2(16,591,260–20,473,937)x3	3.882	68	RAI	Slender build, DD, SLD, DIM, Aut and FD	F/−	ND		Potocki-Lupski syndrome
#236	Dup	arr[hg19] 17q23.3q24.2(62,339,243–65,959,327)x3	3.620	31	BPTF, PSMD12	Slender build, DD, behavioral disorder, FD, Microcephaly and Cardiomyopathy	F/−	ND		—
#237	Del	arr[hg19] 13q21.32q32.1(65,840,171–95,798,028)x1	29.958	74	—	Not reported	F/−	ND		partial 13q monosomy syndrome
#238	Del	arr[hg19] 7p14.1p12.3(41,339,411–47,849,443)x1	6.510	59	GLI3	CAs, DD, SLD, ID and FD	F/−	ND		Greig syndrome
#249	Del	arr[hg19] 10q26.11q26.3(121,477,949–135,426,386)x1	13.948	105	—	DD, ID, FD and microcephaly	F/−	ND		10q26 deletion syndrome
#251	Dup	arr[hg19] 19p13.3(260,911–2,328,485)x3	2.068	90	61 OMIM genes	Obesity, DD, ID, FD and ectodermal dysplasia	M/*2Pv	ND		partial trisomy 19p13 syndrome
#251	Del	arr[hg19] 20q13.33(62,288,778–62,913,645)x1	625	32	16 OMIM genes	Obesity, DD, ID, FD and ectodermal dysplasia	M/*2Pv	ND		—
#255	Dup	arr[hg19] 22q11.21q11.23(18,493,187–24,313,652)x3	5.820	125	TBX1	DD, Aut and FD	M/*VOUS	ND		22q11.21 duplication syndrome
#263	Del	arr[hg19] 9p24.2p22.2(4,339,192-18,272,756)x1	13.934	45	32 OMIMs	Hypotonia, CAs, DD and FD	M/−	ND	47, XY + mar	9p deletion syndrome
#264	Dup	arr[hg19] 1q21.1q21.2(146,496,425–147,819,815)x3	1.323	16	PRKAB2, FMO, CHD1L, GJA5, GJA8, GPR89B	Hyperactivity, behavioral disorder, SLD, ASD, LD	M/−	ND		1q21.1 duplication syndrome
#270	Del	arr[hg19] 15q11.2(22,770,421–23,282,799)x1	512	8	NIPA1, NIPA2, CYF1P1, TUBGCP5	Convulsions, CAs, DD, epilepsy, hearing deficit, FD and hirsutism	M/*3Pv	ND		15q11.2 BP1–BP2 microdeletion syndrome (OMIM 615656)
#270	Del	arr[hg19] 18q22.1q23(65,997,926–78,014,123)x1	12.016	42	RTTN, MBPP, TSHZ1	Convulsions, CAs, DD, epilepsy, hearing deficit, FD and hirsutism	M/*3Pv	ND		—
#270	Dup	arr[hg19] 3q26.32q29(178,907,147–197,851,986)x3	18.945	180	112 OMIMs	Convulsions, CAs, DD, epilepsy, hearing deficit, FD and hirsutism	M/*3Pv	ND		—
#296	Del	arr[hg19] 16p11.2(29,580,020–30,176,508)x1	596	28	ALDOA	Obesity, convulsions, DD, SLD, LDO., ID, epilepsy and hypoglycemia	M/−	ND		16p11.2 deletion syndrome
#305	Dup	arr[hg19] Xq27.3q28(142,412,280–155,233,098)x2	12.821	167	FMR1-AS1, FMR1, AFF2, MECP2	Obesity, CAs, DD, ID, FD and cutis marmorata	M/−	ND	46, XY, add(X)(p22)	Xq27.3-q28 and Xq28 duplication syndrome
#306	Dup	arr[hg19] 15q11.2q13.1(23,286,571–28,946,433)x4	5.660	116	CYF1P1, PWRN1, PWRN2, SNRPM, UBE3A	DD	M/−	ND		15q11-q13 duplication syndrome
#312	Del	arr[hg19] 22q11.21(18,916,842–21,798,907)x1	2.882	70	TBX1	CAs, SLD, LDO. and FD	M/−	ND		Di George syndrome
#331	Del	arr[hg19] 4p16.3(68,345–964,416)x1	896	18	LETM1, WHSC1	DD, Epilepsy and FD	M/*2Pv/*VOUS	ND		Wolf-Hirschhorn syndrome
#331	Del	arr[hg19] 4p16.3(970,878–4,015,580)x1	3.045	50	NSG1	DD, Epilepsy and FD	M/*2Pv/*VOUS	ND		—
#332	Dup	arr[hg19] 3q29(192,443,188–197,851,986)x3	5.409	70	35 OMINs	DD, ID and FD	F/*2Pv	ND		3q29 Microduplication syndrome
#332	Del	arr[hg19] 7q34q36.3(143,069,244–159,119,707)x1	16.050	142	67 OMIMs	DD, ID and FD	F/*2Pv	ND		7q34-36 deletion syndrome
#339	Del	arr[hg19] 12p13.2p13.1(10,922,516–12,937,320)x1	2.015	40	LRP6	Slender build, FD, and alopecia	F/−	ND	46, XX, Inv (12)(p13q24.1)	—
#343	Del	arr[hg19] 1p36.33p36.31(849,466–5,830,248)x1	4.981	94	GABRD, PRKC2, SKI,	DD, SLD, ID, CAs and hypothyroidism	F/−	ND		1p36 deletion syndrome
#345	Del	arr[hg19] 14q32.2q32.31(100,095,248–102,755,064)x1	2.660	117	PEGS (DLK1 and RTL1), MEGS (MEG3 and MEG8)	Low weight, short stature, prematurity, IUGR, ataxia, scoliosis, DD, SLD, SID, Aut, FD and early puberty	F/−	ND		Temple syndrome
#366	Del	arr[hg19] 6q25.1q26(150,944,729–164,003,180)x1	13.058	71	ARID1B	Low weight, short stature, CAs, DD, FD and ventricular septal defect	F/−	ND		6q25.1 deletion syndrome
#372	Dup	arr[hg19] 4p16.3p16.1(68,345–9,509,606)x3	9.441	148	72 OMIMs	Hypotonia, DD, SLD, LDO., DIL and behavioral disorder	M/*2Pv	ND		—
#372	Del	arr[hg19] 8p23.3p23.1(158,048–6,938,050)x1	6.780	46	MCPH1	Hypotonia, DD, SLD, LDO, DIL and behavioral disorder	M/*2Pv	ND		—
#377	Dup	arr[hg19] 22q11.21(18,648,855–21,461,017)x3	2.812	69	TBX1	Convulsions, ID, DD, SLD, ADHD and FD	M/−	ND		22q11.21 duplication syndrome
#385	Del	arr[hg19] 21q22.12q22.2(35,834,713–39,831,660)x1	3.997	32	DYRK1A	Convulsions, ID, DD, SLD, Aut, cardiomyopathy, abnormal external genitalia and thrombocytopenia	M/−	ND		21q22.12 microdeletion syndrome
#392	Dup	arr[hg19] 21q11.2q22.3(15,006,457–44,968,648)x3	29.962	224	—	Not reported	M/*3Pv	ND	46, XY, r(21)(p21q22.3)[?]/46, XY, idic(21)(p13)[?]	trisomy of chromosome 21
#392	Dup	arr[hg19] 21q22.3(44,974,017–45,685,800)x3	711	12	—	Not reported	M/*3Pv	ND	46, XY, r(21)(p21q22.3)[?]/46, XY, idic(21)(p13)[?]	—
#392	Del	arr[hg19] 21q22.3(45,685,800–48,097,372)x1	2.411	58	—	Not reported	M/*3Pv	ND	46, XY, r(21)(p21q22.3)[?]/46, XY, idic(21)(p13)[?]	Terminal 21q del
#399	Dup	arr[hg19] 17p11.2(16,591,260–20,462,723)x3	3.871	69	RAI	Short stature, DD, FD and macrocephaly	F/−	ND		Potocki-Lupski syndrome
#407	Del	arr[hg19] 21q22.3(45,434,816–48,093,361)x1	2.659	63	—	Low weight, abnormal growth, convulsions, neuropathies, DD, FD and congenital cardiopathy	F/*2Pv	ND		—
#407	Dup	arr[hg19] 3q26.1q29(166,855,496–197,851,444)x3	30.996	228	SHOX2	Low weight, abnormal growth, convulsions, neuropathies, DD, FD and congenital cardiopathy	F/*2Pv	ND	46, XX, add(21)(q22.3)	Distal trisomy 3q
#409	Del	arr[hg19] 22q11.21(18,916,842–20,716,903)x1	1.800	46	PRODH, TBX1, DGCR6L	CAs, ligament laxity, DD and FD	M/−	ND		Di George syndrome
#416	Del	arr[hg19] 18q21.32q23(58,921,746–78,013,728)x1	19.092	75	PIGN	Obesity, CAs, DD, ID, deafness, Aut, FD and thrombocytopenia	M/−	ND		18 q21.32-qter deletion syndrome
#422	Dup	arr[hg19] 18p11.32p11.21(136,227–15,099,116)x4	14.963	88	46 OMIMs	CAs, DD, FD, macrocephaly and renal cysts	M/karyotype 47, XY +mar(30)	ND	47, XY + mar	tetrasomy 18p11.21-p11.32
#433	Dup	arr[hg19] 7q31.32q33(122,736,512–136,162,906)x3	13.426	101	LEP	ID	M/−	ND		partial trisomy 7q31.32q33
#443	Dup	arr[hg19] 22q12.3q13.1(35,888,588–38,692,765)x4	2.804	59	45 OMIMs	Low weight, short stature, DD, SLD, Aut, behavioral disorder, FD and mongolian stains	M/−	ND		—
#445	Dup	arr[hg19] 5p14.3–p15.31 (6,801,589–18,992,827)x3	12.131	—	—	Not reported	Brother of #149	46, XY, t(1; 2)(q44;~p23-pter); t(5; 7)(p14.3-p15.31; p22) pat.		partial trisomy 5p14.3-p15.31

Pathogenic CNVs found by CMA in the cohort, with the number of genes present in the region, listing the most relevant genes and phenotypes for each individual. Du p = Duplication, Del = Deletion, CA = congenital anomalies, DD = developmental delay, MID = mild intellectual disability, SID = severe intellectual disability, Aut = autism, Mot Dif = motor difficulties, FD = facial dysmorphisms, SLD = speech and/or language delay or impairment, IUGR = intrauterine growth restriction, ADHD = Attention-deficit/hyperactivity disorder, LDO = learning difficulty only, LD = Learning disability, ND = not determined. F = Female, M = Male. *VOUS = Patients with VOUS (CNV). *2**Pv** = Patients with 2 pathogenic CNVs. *3**Pv** = Patients with 3 pathogenic CNVs.

**Figure 2:**
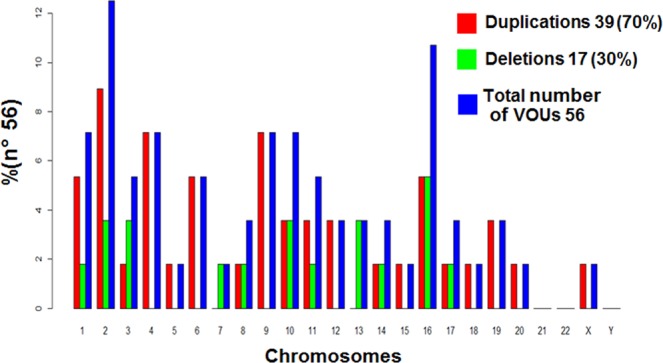
VOUS per chromosome.

Variants of uncertain significance (VOUS), which also are rare CNVs, were the main findings in 12% (49/420) of the patients, summing up a total 56 CNVs, 17 deletions and 39 duplications, (Table 3). These variants were found on most chromosomes except for 21, 22 and Y, and contained from 1 to 48 genes (SD = 10:19, Mo = 4), of which from 1 to 28 (SD = 5.06 Mo = 2) are genes cited in the OMIM database (OMIM genes). Figure 2 illustrates the frequency and amount of VOUS per chromosome.

**Table 3:** VOUS found in the cohort.

Case	Type	Microarray Nomenclature	Size (Kbp)	N° of Genes	N° of Genes in OMIM	Important Genes	Phenotype	Gender/Notes
#1	Dup	arr[hg19] 2p24.1(23,982,758-24,813,485)x3	831	18	7	ATAD2B, UBXN2A	MID, overweight	F
#5	Dup	arr[hg19] 6q15(89,917,335-90,485,874)x3	568	7	4	GABRR1, GABRR2	MID, Mot Dif and hyperactivity	M
#6	Dup	arr[hg19] 2q37.2q37.3(236,733,535-237,355,774)x3	622	4	2	AGAP1, GBX2	DD, convulsions and FD	F
#7	Dup	arr[hg19] 1q44(246,324,898-246,688,599)x3	363	2	1	SMYD3	Aut, Mot Dif, convulsions and FD	M
#13	Del	arr[hg19] 11q14.1(84,050,388-84,415,990)x1	365	1	1	DLG2	Aut, LDO, Mot Dif, FD and SLD	M
#19	Dup	arr[hg19] 8q21.13(82,061,218-84,515,685)x4	2.454	10	6	IMPA1	DD, FD, gastroschisis bladder exstrophy, hydronephrosis and Abnormal growth	M/Affected brother (#18)
#21	Del	arr[hg19] 2q13(110,504,318-111,365,996)x1	861	16	3	NPHP1	ID	M
#32	Dup	arr[hg19] 20q13.33 (61,854,236-62,054,955)x3	200	9	5	KCNQ2, CHRNA1	Convulsions, low weight, prematurity, FD, microcephaly and tracheoesophageal fistula	M
#40	Del	arr[hg19] 14q24.2(73,590,938-73,776,190)x1	185	4	2	PSEN1, NUMBP1	Aut and SLD	M
#43	Del	arr[hg19] 16q23.2(80,260,131-80,701,060)x1	440	2	1	DYNLRB2, CDYL2	MID, Aut, Mot Dif, SLD, hyperactivity and FD	M
#50	Del	arr[hg19] 13q12.12(60,425,635-60,688,042)x1	262	25	2	SGCG, SACS	MID	F
#58	Dup	arr[hg19] 11q22.3(102,946,063-103,827,049)x3	880	4	2	DYNC2H1	DD, LDO, Mot Dif and FD	M
#64	Dup	arr[hg19] 9q34.3(139,381,821-140,086,032)x3	704	48	28	NOTCH1	DD, SLD, ID and FD	M
#81	Dup	arr[hg19] 16p13.3(549,826-1,449,862)x3	900	45	26	CACNA1H	SLD, convulsions and FD	M/*Pv
#82	Dup	arr[hg19] 4q35.2(188,106,543-189,797,261)x3	1.691	5	1	ZFP42	DD and SLD	M
#86	Del	arr[hg19] 13q21.2(60,425,635-60,688,042)x1	262	2	1	DIAPH3	DD and LDO	F
#89	Dup	arr[hg19] 9p24.3(319,876-517,446)x3	198	2	2	DOCK8, KANK1	Aut, SLD, mot dif and FD	F
#109	Dup	arr[hg19] 4q31.1(139,758,054-139,988,340)x3	230	2	1	CCRN4L	DD and FD	M
#112	Dup	arr[hg19] 9p13.3(34,211,157-34,395,294)x3	184	5	3	UBAP1, NUDT2	SID, Aut, convulsions, SLD, mot dif and FD	M
#117	Dup	arr[hg19] 19q13.33(48,206,212-48,431,081)x3	224	25	7	CORD2	Short stature, abnormal brain structure, CAs, DD, FD, hirsutism and anemia	F/−
#136	Dup	arr[hg19] 4q28.1q28.2(128,789,028-128,891,808)x3	103	3	2	PLK4	Low weight, short stature, IUGR, FD, thin hair, and skin spots - no ID	F/−
#138	Dup	arr[hg19] 6p21.2(37,609,169-37,868,513)x3	260	2	2	MDGA1	Prematurity, DD, polydactyly, aggression, FD, difficulties of swallowing food, vomiting with fatty food, cutis marmorata, microcytic and hypochromic anemia	M/−
#144	Del	arr[hg19] 8q13.1q13.2(67,999,679-68,190,627)x1	191	2	2	CSPP1	DD, SLD, ID and FD	F/−
#178	Dup	arr[hg19] 11q23.3(117,000,284-117,312,611)x3	312	10	7	DSCAML1, CEP164, BACE1	Slender build, DD, Aut, FD, macrocephaly	M/−
#180	Del	arr[hg19] 16p13.3(6,243,228-6,835,898)x1	593	1	1	RBFOX1	DD, hypothyroidism	M/−
#215	Del	arr[hg19] 3q26.33(179,508,262-179,621,954)x1	114	1	1	PEX5L	Motor Delay, DD, ID, Aut and ADHD	M/−
#223	Dup	arr[hg19] 15q24.1(72,838,805-73,581,757)x3	743	8	4	BBS4	Short stature, IUGR, DD, MID and FD	M/*3 V
#223	Dup	arr[hg19] 3p26.3(255,645-1,510,822)x3	1.255	2	2	CTN6, CHL1	Short stature, IUGR, DD, MID and FD	M/*3 V
#223	Dup	arr[hg19] 6q25.3(156,488,875-158,534,725)x3	2.045	9	4	ARID1B, SYNJ2	Short stature, IUGR, DD, MID and FD	M/*3 V
#245	Dup	arr[hg19] 14q12(26,490,666-27,520,832)x3	1.030	2	1	NOVA1	Obesity, encephalopathy, CAs, DD and FD	F/−
#248	Del	arr[hg19] 10q23.1(87,392,282-87,791,684)x1	399	1	1	GRID1	Abnormal brain structure, DD, SLD, FD and microcephaly	M/−
#255	Del	arr[hg19] 10q23.1(87,691,467-87,843,627)x1	152	1	1	GRID1	DD	M/*Pv
#268	Del	arr[hg19] 2q13(110,504,318-111,365,996)x1	861	16	3	NPH1	ASD	M/−
#276	Dup	arr[hg19] Xq26.2(130,672,818-130,967,726)x3	295	2	3	KAL1	DD, FD, cardiomyopathy, thyroid dysfunction and myopia	F/−
#278	Dup	arr[hg19] 19q13.42(54,201,711-54,420,807)x3	219	39	9	MIR, NLRP12	Epilepsy, abnormal brain structure and ID	F/−
#290	Dup	arr[hg19] 2q13(110,496,601-110,983,418)x3	487	14	3	NPHP1	Genetic counseling	M/−
#294	Dup	arr[hg19] 2q13(110,498,141-110,980,295)x3	482	14	3	NPHP1	DD, ID, FD and Congenital cardiopathy	F/−
#299	Dup	arr[hg19] 17q11.2(28,952,286-29,150,025)x3	198	4	1	CRLF3	DD, Aut and Behavioral disorder	M/−
#309	Del	arr[hg19] 17p13.1(6,949,507-7,217,381)x1	268	16	15	-	Short stature, DD, ID, FD and microcephaly	M/−
#311	Dup	arr[hg19] 1p31.3(61,699,736-62,125,970)x3	426	2	1	NFIA	Obesity, CAs, DD, SLD and ID	F/−
#319	Dup	arr[hg19] 16p13.3(1,252,411-1,404,818)x3	152	9	8	5 OMIMs	Anal imperforation, onfalocele and cloacal exstrophy	F/−
#331	Dup	arr[hg19] 4p16.3p16.2(4,025,257-4,618,896)x3	594	7	3	NSG1	DD, epilepsy and FD	M/*Pv
#336	Dup	arr[hg19] 1q25.3(183,589,206-183,827,325)x3	238	3	3	ARPC5, APOBEC4, RGL1	DD and FD	F/−
#342	Del	arr[hg19] 3p24.2(24,376,230-24,492,572)x1	117	1	1	THRB	DD, Bilateral hearing impairment and FD	F/−
#346	Del	arr[hg19] 7q31.1(111,485,313-111,922,531)x1	437	2	2	DOCK4	Low weight, slender build, motor delay, DD, SLD, SID and Aut.	M/−
#354	Dup	arr[hg19] 9q33.1(118,409,943-119,207,073)x3	797	4	3	NOC2L	Consanguineous parents, quadriparesis, DD, FD and ostium secundum	M/−
#359	Dup	arr[hg19] 5q14.1(80,019,759-80,535,750)x3	516	6	3	MSH3, RASGRF2, CKMT2	Convulsions, LDO, MID and behavioral disorder	F/−
#360	Del	arr[hg19] 1p31.1(72,257,666-72,499,784)x1	242	2	1	NEGR1	Convulsions, LDO, F MID and hearing loss	F/−
#369	Dup	arr[hg19] 12p11.22p11.21(30,175,955-31,570,927)x3	1.394	9	3	IPO8, CAPRIN2, DDX11	Abnormal brain structure and DD	M/−
#383	Dup	arr[hg19] 10q11.23(51,250,417-51,755,110)x3	505	7	4	PARG, MSMBP, NCOA4, TIMM23	Convulsions, DD, SLD, Aut., Behavioral disorder and gluten intolerance	M/−
#384	Dup	arr[hg19] 10q21.1(59,984,568-60,285,875)x3	301	5	5	IPMK, CISD1, UBE2D1, TFAM	Motor delay and chronic encephalopathy	M/−
#384	Del	arr[hg19] 16p13.3(7,108,169-7,225,285)x1	117	1	1	RBFOX1	Motor delay and chronic encephalopathy	M/−
#384	Dup	arr[hg19] 18q22.3(72,755,482-73,023,597)x3	268	3	1	TSHZ1	Motor delay and chronic encephalopathy	M/−
#397	Dup	arr[hg19] 16p12.2(21,817,921-22,431,357)x3	613	9	3	UQCRC2, EEF2K, CDR2	DD, Aut and FD	M/−
#401	Dup	arr[hg19] 2q11.1(95,733,867-96,279,208)x3	545	8	3	ZNF2, MRPS5, KCNIP3	Autism	F/−
#423	Dup	arr[hg19] 12q21.31(80,559,698-80,918,615)x3	358	2	2	OTOGL, PTPRQ	CAs, ID and FD	F/−
#444	Del	arr[hg19] 16p13.3(6,644,079-6,675,606)x1	31	1	1	RBFOX1	ASD	M/−

Variants of unknown significance found in the cohort, with the number of genes present in the region, listing the most relevant genes and phenotypes for each individual. Dup = Duplication, Del = Deletion, CA = Congenital Anomalies, DD = Developmental Delay, ID = Unspecified intellectual disability, MID = Mild Intellectual Disability, MID = Moderate Intellectual Disability, SID = Severe Intellectual Disability, Aut = Autism, Mot Dif = Motor Difficulty and FD = Facial Dysmorphisms, SLD = speech and/or language delay or impairment, IUGR = Intrauterine growth restriction, ADHD = Attention-deficit/hyperactivity disorder, LDO = learning difficulty only, ASD = Autism spectrum disorder, F = Female, M = Male. *3 V = Patients with 3 VOUS (CNV). Pv = Patients with pathogenic CNV. *2Pvp = Patients with 2 pathogenic CNVs.

Four of these VOUS (in cases #180, #223, #384 and #444) are discussed in greater detail, because they were considered potentially pathogenic, however with no compelling evidence at this point (Table 4).

**Table 4:** CNVs Subclassified VOUS as potentially pathogenic VOUS.

Case	Type	Microarray Nomenclature	Size (Kbp)	N° of Genes	N° of Genes in OMIM	Important Genes	Phenotype	Gender/Notes
#180	Del	arr[hg19] 16p13.3(6,243,228-6,835,898)x1	593	1	1	RBFOX1	DD, Hypothyroidism	M/−
#223	Dup	arr[hg19] 15q24.1(72,838,805-73,581,757)x3	743	8	4	BBS4	Short stature, IUGR, DD, MID, FD, dolichocephaly, high-arched palate, microtia, breast hypertelorism and constipation	M/*3V
#223	Dup	arr[hg19] 3p26.3(255,645-1,510,822)x3	1.255	2	2	CTN6, CHL1	Short stature, IUGR, DD, MID, FD, dolichocephaly, high-arched palate, microtia, breast hypertelorism and constipation	M/*3V
#223	Dup	arr[hg19] 6q25.3(156,488,875-158,534,725)x3	2.045	9	4	SNX9, ARID1B	Short stature, IUGR, DD, MID, FD, dolichocephaly, high-arched palate, microtia, breast hypertelorism and constipation	M/*3V
#384	Del	arr[hg19] 16p13.3(7,108,169-7,225,285)x1	117	1	1	RBFOX1	Mot Dif, Chronic Encephalopathy and spastic quadriparesis	M/−
#444	Del	arr[hg19] 16p13.3(6,644,079-6,675,606)x1	31	1	1	RBFOX1	DD and ASD	M/−

Variants of unknown significance with potential pathogenicity, found in the cohort, with the number of genes present in the region, listing the most relevant genes and phenotypes for each individual. Dup = Duplication, Del = Deletion, IUGR = Intrauterine growth restriction, DD = Developmental Delay, MID = Mild Intellectual Disability, FD = Facial Dysmorphisms, Mot Dif = Motor Difficulty, ASD = Autism spectrum disorder, F = Female, M = Male. *3 V = Patients with 3 VOUS (CNV).

All other CNVs were interpreted as benign or as common genetic polymorphisms. In 70% of the cases, they were the only findings present in the genome of a patient, and thus considered a negative result for clinically relevant CNVs.

**Figure 3:**
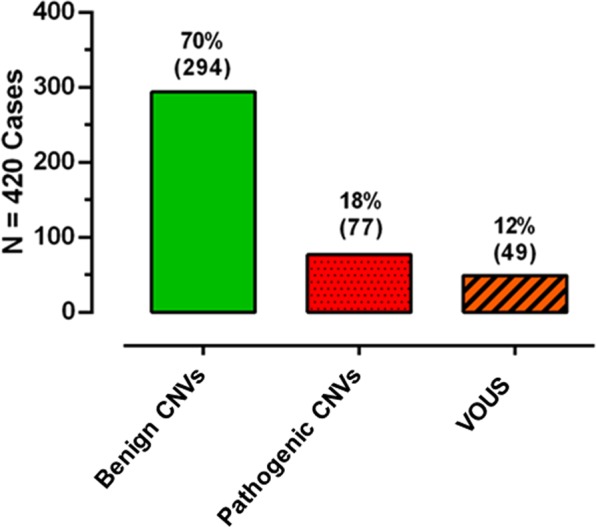
Classification of cases per most relevant CNV found.

Figure 3 Patients grouped according to the most relevant CNV found in their genomes.

### Phenotypic characterization

**Figure 4:**
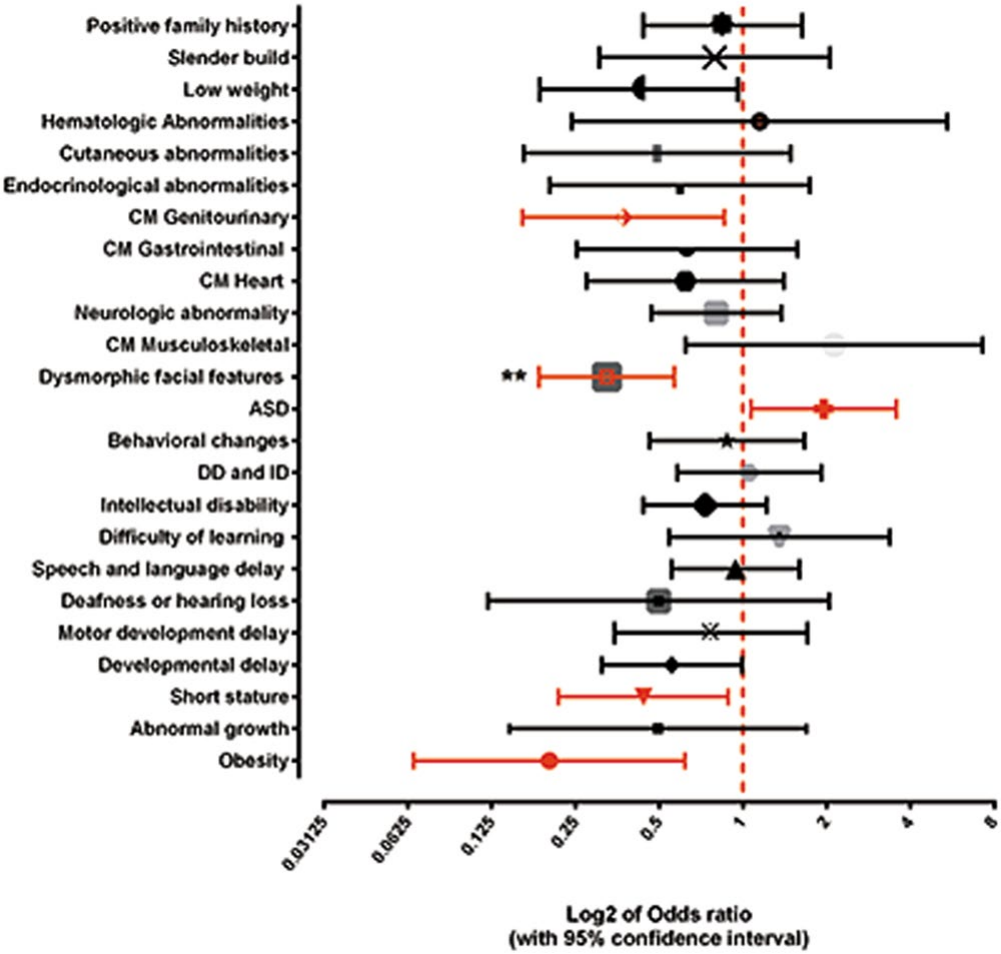
Odds ratios of pathogenic CNVs in cohort study patients. Odds ratios shown in log2 scale. Odds ratios with a p-value < 0.05, two tailed were displayed in red, while others were shown in black. **p-value < 0.001. CM: Congenital malformations, ID: Intellectual disability; DD: Developmental delay and ASD: Autism spectrum disorder.

Of the 420 cases, three were not included in the phenotypic characterization because it was not possible to obtain clinical data. The features registered in our cohort are listed in Table 5. Most patients, besides the main reasons of referral (DD, ID, ASD) had additional characteristics, including dysmorphologies, psychiatric or behavioral issues, or variations in height or body weight, whose relation to the main problem often is unclear. Many have syndromic features, as can be concluded by the high presence of congenital abnormalities and atypical facial appearance. As expected, 80% of the individuals of the studied cohort had DD/ID (the main reasons for referral). DD and ID are cited here together because ID is only diagnosed above 5 years of age, however it is a known fact that most individuals with DD in early infancy will later be diagnosed with ID. Of the patients in our study 67% had DD at the time of the study or at an earlier age, with 41% considered intellectually disabled. Facial dysmorphisms (most of them minor) were reported for 53% and ASD for 32%. Other phenotypes were in lower frequencies. Univariate analysis (chi-square or Fisher’s test when more appropriate) indicated predictive phenotypes for a higher diagnostic result (a higher chance to have a pathogenic CNV) in our cohort with ND: dysmorphic facial features (*p*-value = <0.0001, OR = 0.32), obesity (*p*-value = 0.006, OR = 0.20), short stature (*p*-value = 0.032, OR = 0.44), genitourinary anomalies (*p*-value = 0.032, OR = 0.63) and ASD (*p*-value = 0.039, OR = 1.94) (Fig. 4). There was no significant higher diagnostic result by CMA for the other phenotypes.

**Table 5:** The clinical characteristics recorded for patients with negative and pathogenic CMA results.

Signs/Symptoms	In the cohort (N = 417)	Negative (N = 295)^#^	Pathogenic (N = 75)^#^	*p*-value	Odds ratio
**CHARACTERISTICS**					
Obesity	4% (16/417)	2% (6)	9% (7)	0.006^*f^	0.20
Low weight	7.5% (31/417)	6% (18)	13% (10)	0.061	0.42
Abnormal growth	3% (12/417)	3% (8)	5% (4)	0.277^f^	0.49
Short stature	11% (45/417)	9% (27)	19% (14)	0.032^*^	0.44
Slender build	7% (30/417)	6% (19)	8% (6)	0.823	0.79
Prenatal problems	6% (25/417)	—	—		
Positive family history	18% (75/417)	16% (48)	19% (14)	0.074	0.84
of Intellectual disability or developmental delay	13% (54/417)	—	—	—	
of Congenital anomalies	6.5% (27/417)	—	—	—	
of Psychiatric disorder	7% (30/417)	—	—	—	
**NEURODEVELOPMENT**		—	—	—	
Developmental delay	67% (281/417)	64% (188)	76% (57)	0.061	0.55
Motor development delay	11% (47/417)	9% (28)	12% (9)	0.666	0.76
Deafness or hearing loss	2% (10/417)	2% (6)	4% (3)	0.394^f^	0.49
Speech and language delay and/or dyslalia	33% (137/417)	35% (102)	36% (27)	0.924	0.93
Difficulty of learning	10% (44/417)	10% (31)	8% (6)	0.667	1.34
Intellectual disability	41% (171/417)	39% (115)	47% (35)	0.280	0.73
Mild	6% (26/417)	—	—		
Moderate	2% (10/417)	—	—		
Severe	4% (18/417)	—	—		
Not Specified	28% (117/417)	—	—		
Intellectual disability and/or developmental delay	80% (334/417)	77% (227)	76% (57)	0,983	1.05
**BEHAVIORAL**		—	—		
Behavioral changes (Obsessive-compulsive disorder, attention deficit hyperactivity disorder, self and hetero-aggression, behavior disorder, psychosis)	19% (78/417)	18% (53)	20% (15)	0.811	0.876
Autism Spectrum Disorder	32% (134/417)	35% (102)	21% (16)	0.039*	1.94
Syndromic Autism	11% (44/417)	—	—		
Asperger Syndrome	2% (7/417)	—	—		
Non-Syndromic Autism	20% (83/417)	—	—		
**CONGENITAL MALFORMATION(S) AND/OR DYSMORPHISM(S)**	58.5% (244/417)	—	—		
**FACIAL MALFORMATIONS/DYSMORPHISMS**	53% (222/417)	47% (139)	73% (55)	<0,0001*	0,32
Long face	2% (10/417)	—	—		
Wide face	0% (1/417)	—	—		
Narrow face	1% (4/417)	—	—		
Triangular face	1% (3/417)	—	—		
Asymetrical face	2% (9/417)	—	—		
Cleft palate	3% (12/417)	—	—		
Micrognathia	3% (13/417)	—	—		
Mouth/Lips (unusual)	5% (21/417)	—	—		
Microcephaly (Craniosynostosis included)	8% (33/417)	—	—		
Macrocephaly	3% (13/417)	—	—		
Ears (dysmorphic)	11% (46/417)	—	—		
Eyes (unusual)	16% (68/417)	—	—		
Forehead (unusual)	2% (7/417)	—	—		
Eyebrows (unusual)	2% (7/417)	—	—		
Nose (unusual)	5% (25/417)	—	—		
Hair (unusual)	2% (10/417)	—	—		
Not Specified	18% (76/417)	—	—		
**OTHER CONGENITAL MALFORMATIONS**		—	—		
Musculoskeletal (scoliosis, diaphragmatic hernia, vertebral anomaly)	19% (78/417)	8% (24)	4% (3)	0.326	2.12
Upper limb anomalies	8% (33/417)	7% (22)	8% (6)	1	0.92
Lower limb anomalies	7% (30/417)	9% (27)	13% (10)	0.388	0.65
Heart anomalies and malformations	9% (36/417)	8% (23)	12% (9)	0.354	0.62
Gastrointestinal anomalies and malformations	8% (34/417)	6% (18)	9% (7)	0.460	0.63
Genitourinary anomalies and malformations	6% (25/417)	5% (16)	13% (10)	0.032^*^	0.37
**NEUROLOGIC ABNORMALITY**	37% (155/417)	30% (88)	35% (26)	0.502	0.80
Epilepsy and/or seizures	15% (61/417)	—	—		
Ataxia	2% (9/417)	—	—		
Hypotonia	8% (32/417)	—	—		
Abnormal brain structure	13% (53/417)	—	—		
Endocrinological abnormalities	5% (23/417)	4% (12)	7% (5)	0.354^f^	0.59
**CUTANEOUS ABNORMALITIES** (hyper and hypopigmentation, hemangioma, freckles, café-au-lait spots and others)	4% (19/417)	3% (10)	7% (5)	0.198^f^	0.49
**HEMATOLOGIC ABNORMALITIES**	3% (14/417)	3% (9)	3% (2)	1^f^	1.14

^#^Patients with VOUS as most relevant CNV found were excluded from the correlation, because they represent inconclusive diagnosis. *Significant statistical correlation found between pathogenic CNV and phenotype. ^f^In phenotypes with n < 20, Fisher’s test was more appropriate.

Table 5 summarizes the clinical features recorded for patients with negative and positive CMA results with the percentage (and number) of patients presenting them. Most patients have more than one relevant phenotype.

### Classical karyotyping and CMA

Seventeen patients informed previous abnormal karyotyping results (Table 6), three of which are not very understandable or with a question mark (#282, #412 and #430). For 12 cases, CMA specified the sequences involved, often with unexpected findings, hinting to the mechanism of occurrence of the anomaly and explaining phenotypes that the karyotype by itself suggested otherwise. In case #196, for instance, CMA identified a deletion in the short arm of chromosome 5, whereas the chromosomal analysis of the patient (46, XX, 5p+) indicated additional DNA in chromosome 5. CMA revealed also that the additional DNA in chromosome 5 originated from a partial duplication of the long arm of chromosome 18. For another case, #263 (47, XY +mar), a large deletion was found instead of a gain. Regarding the five cases where the cytogenetic analysis was abnormal and no pathogenic CNV was identified, in one (#138) a VOUS with no apparent relation to the chromosome analysis result was found whereas the other four had a normal CMA result, including the three cases whose informed karyotype was followed by a question mark, indicating that the chromosomal analysis was not conclusive (Table 6).

**Table 6:** Cases with previous abnormal chromosomal results.

case	Karyotype	CMA arr[hg19]	Size (Kbp)	Interpretation	Notes
#44	46, XX, del(22)(q13)	22q13.2q13.33(43,600,479-51,197,766)x1	7.597	Phelan-McDermid Syndrome	As expected, CMA showed a deletion in chromosome 22, where the sequence involved was clarified.
#56	XY,46, del(8)(p21-p11)	8p21.1p11.21(28,393,484-41,026,001)x1	12.632	8p11.2 deletion syndrome	As expected, CMA showed a deletion in chromosome 8, where the sequence involved was clarified.
#116	46, XY, add(22q)	Xq26.3q28(135,224,845-155,233,098)x2	20.008	Region includes Xq26.3, Xq27.3-q28 and Xq28 duplication syndromes	CMA showed that the DNA added to chromosome 12 derived from the terminal part of chromosome Xq.
#127	46, XX, add(18)(q23)	10q25.1q26.3(108,553,165-135,427,143)x3	26.873	Distal trisomy 10q syndrome and Distal 18q deletion syndrome	CMA showed that the additional DNA in chromosome 18 is derived from chromosome 10q, probably as result of an unbalanced t(18,10), causing also deletion of the terminal part of 18q. It is possible that one of the parents is an equilibrated carrier of the translocation.
		18q22.3q23(69,055,745-78,014,123)x1	8.958		
#196	46, XX,5p+	18q21.2q22.1(49,094,563-66,586,144)x3	17.492	Distal trisomy 18q *Cri du Chat* syndrome	CMA showed that the additional DNA in chromosome 5 is derived from chromosome 18q, probably as result of an unbalanced t(5,18), causing also a large deletion of the terminal part of 5p. It is possible that one of the parents is an equilibrated carrier of the translocation.
		18q22.1q23(66,593,317-78,014,123)x3	11.421		
		5p15.33p15.2(113,576-12,747,875)x1	12.634		
#219	46, XX, add(8)(p23.1)	8p23.1p11.22(11,935,023-39,246,760)x3	27.311	8p inverted duplication/deletion [invdupdel(8p)] syndrome	CMA showed that the additional DNA in chromosome 8 is indeed from the same chromosome and also a deletion in 8p occurred, characterizing the 8p inverted duplication/deletion syndrome.
		8p11.22p11.21(39,388,765-42,335,424)x3	6.782		
		8p23.3p23.1(158,048-6,940,661)x1	3.882		
#263	47, XY +mar	9p24.2p22.2(4,339,192-18,272,756)x1	13.934	9p deletion syndrome	Unexpectedly the CMA revealed a deletion in chromosome 9, instead of additional DNA for the marker chromosome. Possibly the marker chromosome is satellite DNA.
#305	46, XY, add(X)(p22)	Xq27.3q28(142,412,280-155,233,098)x2	12.821	Region includes Xq27.3-q28 and Xq28 duplication syndromes	A duplication was found, as expected, showing that it refers to the terminal region of the X chromosome itself.
#339	46, XX, Inv (12)(p13q24.1)	12p13.2p13.1(10,922,516-12,937,320)x1	2.015	A pericentromeric inversion with a deletion in chromosome 12	CMA showed that the inversion caused a deletion in 12p13.
#392	46, XY, r(21)(p21q22.3)[?]/46, XY, idic(21)(p13)[?]	21q11.2q22.3(15,006,457-44,968,648)x3	29.962	Trisomy of chromosome 21 with a loss of the distal part of 21q22.3	CMA showed the trisomy of chromosome 21q, 21(11.2q22.3), revealing that the ring chromosome probably is iso21(11.2q22.3), with a deletion in the distal part of 21(22.3q).
		21q22.3(44,974,017-45,685,800)x3	711		
		21q22.3(45,685,800-48,097,372)x1	2.411		
#422	47, XY +mar	18p11.32p11.21(136,227-15,099,116)x4	14.963	Tetrasomy 18p	CMA revealed that the marker chromosome is an isochromosome 18p.
#407	46, XX, add(21)(q22.3)	3q26.1q29(166,855,496-197,851,444)x3	30.996	3q26.1-q29 duplication syndrome	CMA showed that the additional DNA on chromosome 21 derived from chromosome 3q.
#138	46, XY, del(Yp)[30]	VOUS 6p21.2(37,609,169-37,868,513)x3	259	A small duplication in chromosome 6, considered a VOUS was found.	New karyotyping to clarify previous test would be advisable.
#175	46, XY, t(4; 7) (q31; p14)	Normal CMA result	—	Probably a balanced translocation	One translocation break point possibly disrupted a gene that causes the phenotype. Break-point mapping and sequencing would be advisable.
#282	46, XY, der(10p)? translocation?	Normal CMA result	—	Possibly a balanced translocation. There was a question mark.	New karyotyping to clarify previous test would be advisable.
#412	46, XY, add(13)?	Normal CMA result	—	There was a question mark. Possibly a new karyotyping could give clearer results.	New karyotyping to clarify previous test would be advisable.
#430	46, XX, add(13) PSK?	Normal CMA result	—	There was a question mark. Possibly a new karyotyping could give clearer results.	New karyotyping to clarify previous test would be advisable.

## Discussion

In the present study, a total of 96 pathogenic CNVs were detected in CMA results of 75 patients with ND in the state of Santa Catarina, a diagnostic yield of 18%, within the range of 15–20% diagnostic rate cited in literature for patients with ND in other cohorts^5,9,11–17^. It is important to highlight that the 75 patients with pathogenic CNVs, included 12 patients of the 17 with previous abnormal karyotype result, for whom the CMA test was requested in order to identify the DNA sequences involved. Excluding the 17 cases with known abnormal karyotype results in a diagnostic rate of 15.63%, and when considering only the 122 patients that underwent previous karyotyping and had normal results, the diagnostic rate was not different, 15.57%. However, the diagnostic yield was considered 18% because CMA was essential to uncover the sequences altered in the abnormal karyotype results, and thus was diagnostic, unveiling unexpected findings, like deletions in chromosomes whose karyotype showed additions or deletion when karyotype had suggested addition. These are exemplified by case #127 [46, XX, add(18) (q23)] CMA identified a distal trisomy of 10q with simultaneous distal 18q deletion and for #196 (46, XX, 5p+) CMA revealed a distal trisomy 18q together with a distal deletion in 5p. For case #263 (47, XY +mar), a new chromosomal analysis would be desired, because instead of additional DNA, a large pathogenic deletion in chromosome 9 was found. The CMA results of the 17 cases for whom a previous abnormal chromosomal analysis was reported, are depicted in Table 6, case by case, together with comments about the findings.

Conversely, our results also point to the usefulness of traditional karyotyping to complement the CMA results, allowing an insight into the mechanisms that gave rise to the genetic abnormality, which is relevant for genetic counselling. For instance, from the 15 cases that had more than one rare CNV (pathogenic CNV or VOUS) and no previous abnormal karyotyping, eight involved the terminal region of chromosomes, some of them quite large, combining terminal deletions with terminal duplications, suggesting that they might be derivative chromosomes that arose form a translocation. This can be seen in case #61 (Table 1) with a distal trisomy of chromosome 8q and a simultaneous deletion in the end of the long arm of chromosome 13; #151, with a terminal del18p and a terminal trisomy 7p; #188, with a terminal del21q and a terminal trisomy 19p; #251, with a terminal del20q and a terminal trisomy 19p; #270, with a terminal del18q and a terminal trisomy 3q; #332, with a terminal del7q and a terminal trisomy 3q; #372, with a terminal del8p and a terminal trisomy 4p, and case #407, with a terminal del21q and a terminal trisomy. This derivative chromosome could have been originated during meiosis, during the first mitotic divisions of the zygote or possibly were inherited from a healthy parent that carries the translocation in an equilibrated state. In latter case there is a risk of recurrence for the same or possibly the complementary derivative in another child. Three cases had 2 or 3 CNVs within the same chromosome: case #33, where the microarray result points to a circular chromosome 18, since both ends are deleted; case #331, with two deletions and one duplication, suggesting a del/dup inversion, and case #47, that had two small deletions on the tip of the p arm, surrounding the SHOX gene, indicating a possible del/del inversion including SHOX. Other cases had a combination of interstitial or terminal and interstitial CNVs in two or more chromosomes, pointing to more complex mechanisms.

In 2010, the American College of Medical Genetics recommended CMA as first-tier test for the population of individuals with DD, ID, ASD and multiple congenital anomalies. We agree with that. However, about the often-made question if CMA is a substitute for the classical chromosome analysis or even if CMA is causing karyotyping to be obsolete, we consider that a correct diagnosis requires the combination of CMA and chromosome analysis as stated by others^21^, which observed structural rearrangements in addition to simple deletions or duplications under the microscope in 85 (18%) of 469 cases with an abnormal CMA result. Likewise, chromosome analysis of the parents of individuals with clearly pathogenic terminal deletions/duplications or large CNVs (regardless if terminal or interstitial) should be a follow-up rule, because this knowledge is essential for genetic counselling. For instance, the karyotype of a father of two affected siblings, a girl (#149) with a large deletion in chromosome 5 [5p14.3–p15.31 (6,801,589–18,992,827)] and her brother, #445, with a duplication of the exact same region, revealed complex translocations involving at least four chromosomes, 46, XY, t (1; 2) (q44; ~p23-pter); t(5; 7) (p14.3–p15.31; p22) (Table 1). The genome of this father survived catastrophic events with no obvious clinical consequence for him which, however, left rearrangements (not detectable by MCA) whose deleterious reflexes did affect deeply the development of his two children – in two distinct (or opposite) molecular ways midst an even larger array of possibilities.

Among 17 abnormal karyotypes we had at least one equilibrated translocation, case #175 [46, XY, t(4; 7) (q31; p14)], whose CMA result showed no CNV. This is an interesting case to study because it is unlikely that this translocation has no pathogenic relevance. Possibly the translocation disrupts or interferes with the regulation of the causal gene, which could be identified by breakpoint mapping/sequencing.

The pathogenic CNVs found in this study and the reported phenotypes of the respective patients are detailed in Table 1. It is known that most pathogenic CNVs occur “*de novo*” because of an error during meiotic recombination, an early illegitimate mitotic recombination, or the mutagenic repair of DNA double-strand breaks during the first divisions of the embryonic cells^22^. They can also be consequence of a balanced chromosomal translocation in the genome of one of the parents, therefore classical karyotype test for parents of individuals with large pathogenic CNVs is advisable, since balanced translocations cannot be identified by CMA and there is a high risk of recurrence^23^.

**Figure 5:**
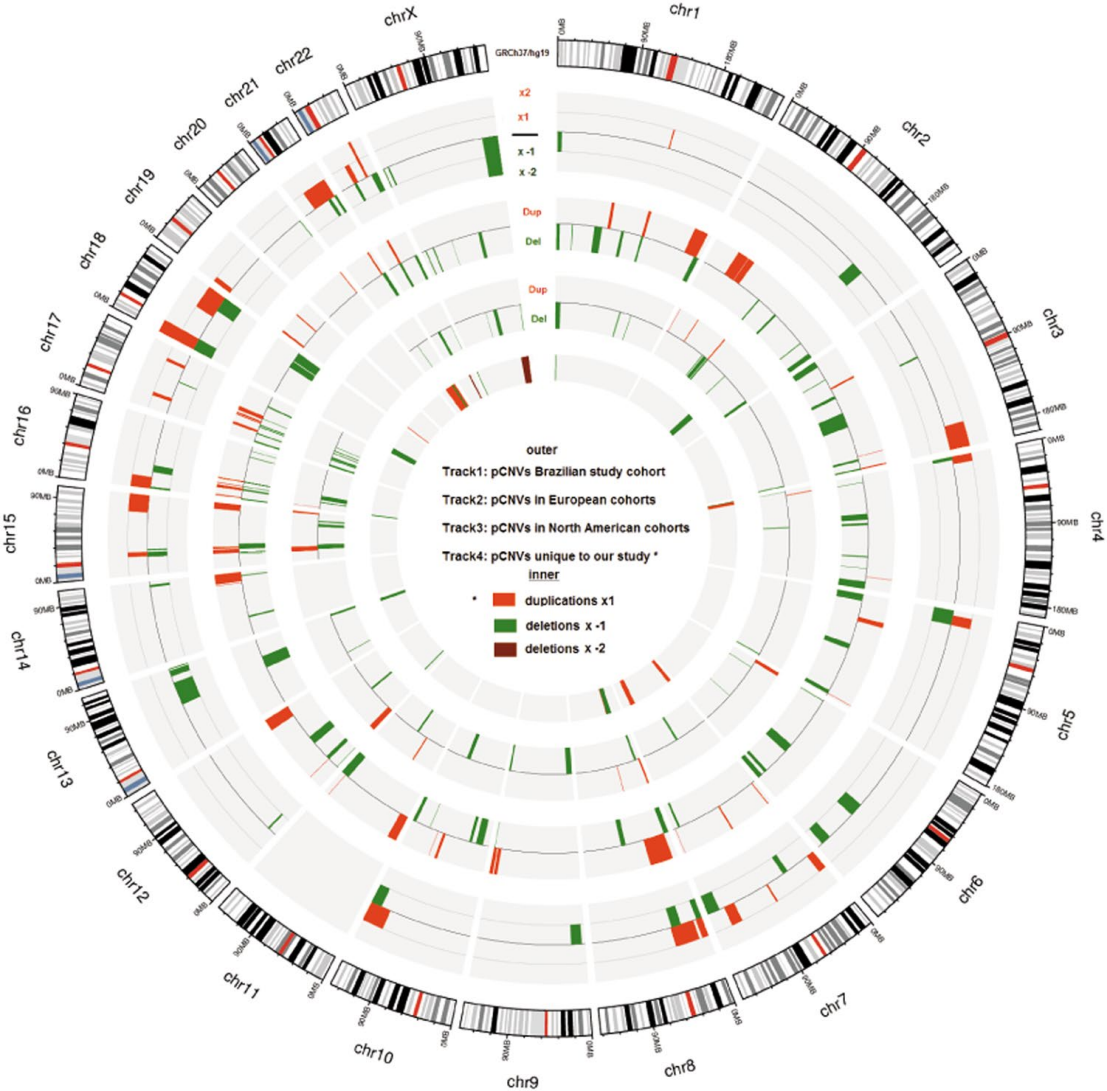
The circle plot compares pathogenic CNVs found in: (first, the outhermost double track) our study of a cohort of 420 individuals with neurodevelopmental disorders (ND), derived from a complex population in the south of Brazil, mostly composed by the Portuguese conquerors, German and Italian immigrants, besides descendants of slaves and of Amerindians; (second double track from the border) studies of 1.245 individuals from five affected European cohorts; (third double track) studies of 15.901 individuals from two affected North American cohorts; (fourth, innermost double track) the pathogenic CNVs detected exclusively in our study, when compared to the other studies in the plot.

We tried to draw comparisons between pathogenic CNVs detected between various studies, which is a challenge, since each study used distinct CMA platforms with probes of varying sizes, densities and characteristics. To allow a comparison, we made a circle plots with the pathogenic CNVs detected in our study together with the pathogenic CNVs detected in cohorts of North America^24,25^ and Europe^13–15,26,27^ using studies that made the data sufficiently available for such analysis (Fig. 5).

Among the studies of the circle plot, the following pathogenic CNVs were detected exclusively in our sample: arr[hg19] 1p36.33p36.32(1,073,574–2,458,606)x1, arr[hg19] 2q31.1-q31.2(174,065,715–190,659,870)x1, arr[hg19] 4p16.3p16.1(68,345–9,509,606)x3, arr[hg19] 4p16.3(68,345–964,416)x1, arr[hg19] 7p22.3p21.3(43,376–9,454,786)x3, arr[hg19] 7q31.32q33(122,736,512–136,162,906)x3, arr[hg19] 8p21.1p11.21(28,393,484-41,026,001)x1, arr[hg19] 8p11.22p11.21(39,388,765–42,335,424)x3, arr[hg19] 12p13.2p13.1(10,922,516–12,937,320)x1, arr[hg19] 13q33.1q34(104,782,510–112,352,804)x1, arr[hg19] 16p13.3(85,880–2,145,951)x1, arr[hg19] 18p11.32p11.31(136,226–4,409,550)x1, arr[hg19] 19p13.3(260,911–1,434,508)x3, arr[hg19] 21q11.2q22.3(15,006,457–44,968,648)x3, arr[hg19] 21q22.12q22.2(35,834,713–39,831,660)x1, arr[hg19] 22q12.3q13.1(35,888,588–38,692,765)x4, arr[hg19] Xp22.3q28(1–247,249,719)x3, arr[hg19] Xp22.33(372,029–578,764)x1, arr[hg19] Xp22.33(679,520–950,907)x1, arr[hg19] Xq26.3q28(135,224,845–155,233,098)x2, arr[hg19] Xq27.3q28(142,412,280–155,233,098)x2, arr[hg19] Xq27.3q28(146,425,635–151,604,987)x2 and arr[hg19] Xq27.3q28(146,418,810–151,604,987)x2.

The interpretation of CNVs is not an absolute science and caution must be used in the report of the results. Palmer *et al*. (2013) already presented data on how the interpretation of CNVs detected by CMA had a significant change over time, with an increase in CNVs classified as pathogenic as new studies and case descriptions are reported. That is why it is important to register the CNVs interpreted as VOUS when no pathogenic CNV is found. In our study we found VOUS (as the most relevant CNV) in 12% (49/420) of the patients in the cohort (Table 2). Although we believe that most of them will have no clinical impact, some of the CNVs in this subgroup possibly will be classified as pathogenic in the future, as more data accumulates. Bellow we highlight four cases where we considered the VOUS potentially pathogenic:

Case #223 = Refers to a boy that was ten years old when he was referred for CMA. He presented short stature, intrauterine growth restriction, DD, mild ID, a narrow face, dolichocephaly, high-arched palate, microtia (small ears), nipple hypertelorism and constipation. His MCA revealed no pathogenic CNV, however three duplication VOUS (Table 4), of which two were considered potentially pathogenic: arr[hg19] 3p26.3(255,645–1,510,822)x3 and arr[hg19] 6q25.3(156,488,875–158,534,725)x3, whose inheritance is unclear. The region arr[hg19] 3p26.3(255,645–1,510,822)x3 duplicates the entire sequence of the contactin 6 gene (CNTN6), LINC01266, a long intergenic ncRNA, and the final of the CHL1 gene (cell adhesion molecule L1 like). CHL1 has been proposed as a candidate gene for intellectual disability of the 3p deletion syndrome^28,29^, and one partial duplication of a similar portion of the CHL1 gene as in case #223 was described, including also the complete CDS of LINC01266, and a small portion of the CNTN6 gene^30^. It is not clear if the partial duplication of CHL1 in was originated by some rearrangement that could have disrupted one of the complete copies of the gene. Contactin 6, encoded by CNTN6 is a neural cell adhesion molecule that has been proposed as one of the critical genes of the 3p deletion syndrome^31^ and deletions or duplications of CNTN6 was suggested to be associated to a wide spectrum of neurodevelopmental disorders^32^. The 6q25.3(156,488,875–158,534,725) genomic region contains the complete sequences of the genes ARID1B (AT-rich interaction domain 1B), TMEM242 (Transmembrane Protein 242), ZDHHC14 (Zinc Finger DHHC-Type Containing 14), SNX9 (Sorting Nexin 9), SYNJ2 (Synaptojanin 2), the beginning of the SERAC (Serine Active Site Containing 1) gene, and the microRNA genes MIR4466 and MIR3692. No complete duplication of any of these genes was found on the DGV. Of those, SYNJ2 is majoritarily expressed in the brain^33^ and is a member of the synaptojanin family, which are key players in the synaptic vesicle recovery at the synapse; TMEM242 is a potential multi-pass membrane protein of unknown function^34^, that is expressed in most tissues^33^**)**, however, with highest expression in the brain; ZDHHC14 is a probable palmitoyltransferase^34^ whose expression is highest in the brain and utherus^33^; SNX9 could involved in several stages of intracellular trafficking and is espressed is most tissues, with very low brain expression^33^ and ARID1B is a component of the SWI/SNF chromatin remodeling complex and its haploinssuficiency is one of the most frequent causes of ID, both, syndromic (Coffin-Siris syndrome) and non-syndromic^35–38^. Coffin-Siris syndrome is characterized by, feeding difficulties in infancy, delayed motor skills, severe speech impairment, mild to severe ID, coarse facial features, hirsutism and its hallmark is the hypoplasia or absence of the 5th distal phalanx of the finger and/or toes. Up to now, only intragenic duplications that probably disrupt gene function were described, however no complete duplication of the gene ARID1B has been described. Duplications comprising the region of chromossome 6 that is duplicated in case #223 are much larger, with the exception of one registered in Decipher, for patient: 287902 with microcephaly and ID, that has a “de novo” duplication of about the same size as the one in our case. Other three duplications including only complete ARID1B alone or with one more gene are also in Decipher, all being the only, or the only non-inherited CNV, found.

Cases #180, #384 and #444 refer to three boys, 4, 2, and 5 years old, respectively, at the date of referral for CMA, because of DD (# 180), motor delay, chronic encephalopathy and spastic quadriparesis (# 384), and DD and ASD (# 444), all of them with a different intragenic deletion in the gene RBFOX1. The RBFOX1 gene (OMIM * 605104), also known as Ataxin-2-binding protein 1 (A2BP1) or FOX1, is one of the largest genes in the human genome and encodes a neuronal RNA binding protein that is highly conserved evolutionarily. It has a very complex transcription unit that generates transcripts from multiple promoters, and presents alternative termination sites. The inclusion of its multiple internal exons is highly regulated, yielding various nuclear and cytoplasmic protein isoforms^39^. In the nucleus, RBFOX1 protein isoforms act as RNA processing factors, while in the cytoplasm they act as proteins that regulate the stability and translation of RNAs involved in cortical development and autism^40,41^.

Changes in RBFOX1 have been related to several neurodevelopmental syndromes, including ID, epilepsy, and ASD^42–44^, with important roles in neuronal migration and synapse network formation during corticogenesis^45^. Specifically, intragenic deletions have been related to neuropsychiatric and neurodevelopmental disorders^42,46,47^.

The case #180 showed a microdeletion 593 Kbp (arr[hg19] 16p13.3(6,243,228–6,835,898)x1), eliminating exon 1 from transcript variant 6 (isoform 4 NM_001142334.1) and exons 2 and 3 from transcripts variants 4, 5 and 7 (respectively, isoform 4 NM_018723.3, isoform 5 NM_001142333.1 and isoform 6 NM_001308117.1).of the gene RBFOX1 which in the reference sequence are non-coding exons of the 5 ‘ region. Besides possibly affecting the transcription of the main isoforms, this microdeletion also affects the promoter of several isoforms of RBFOX1, whose transcription begins after exon two.

Case #384 presented one microdeletion 117 kbp in 16p13.3 (arr[hg19] (7,108,169–7,225,285)x1), involving an intronic region between exon 4 and 5 from transcripts variants 4, 5 and 7 (respectively, isoform 4 NM_018723.3, isoform 5 NM_001142333.1 and isoform 6 NM_001308117.1) and between exon 2 and 3 from from transcript variant 6 (isoform 4 NM_001142334.1) of the RBFOX1 gene, and case #444 had microdeletion of 31 kbp (arr[hg19] 16p13.3(6,644,079–6,675,606)x1) in intron 2 from transcripts variants 4, 5 and 7 (respectively, isoform 4 NM_018723.3, isoform 5 NM_001142333.1 and isoform 6 NM_001308117.1) of the RBFOX1 gene, affecting various isoforms and possibly affecting the isoform promoter region that initiates from transcript variant 6 (isoform 4 NM_001142334.1) after exon 3 of the reference sequence.

It is topic of ongoing discussion of how to communicate the CNVs findings in the reports, where the communication of VOUS is particularly challenging. In clinical practice, it is a confounding factor to have a CNV about which no one can say something for sure. The limitations of the test and, more shockingly, of the current understanding of the results are difficult for the clinician to explain and even more difficult for the patient/guardians to understand. They often cannot settle for the idea that they underwent such an expensive test and the doctors cannot say anything useful or definitive with the results. Even though adequate pre-testing explanation is provided to patients or their guardians, and they sign a consent form which also lists the limitations of the test, for many persons the real understanding of what that means only sinks in after receiving an ambiguous CMA result. It is much easier to explain a negative result that, if not answering the question of why the neurodevelopment was disturbed, at least answers that it is not caused by a genomic imbalance produced by an excess or a deletion of genetic material. A VOUS tends to represent a point of frustration for all involved. The American College of Medical Genetics allows to communicate the likelihood that a VOUS is pathogenic or benign, when well founded in the report and the uncertainty of such classification is clearly communicated. In addition, they also recommend that the report includes guidelines for the continuous monitoring of medical literature, since new knowledge can clarify the CNV’s real clinical impact.

One strategy in the interpretation of a VOUS is to investigate if it occurred “de novo” or has been inherited from one of the parents. Inherited CNVs are more likely benign, whereas “de novo” variants found in ND patients have a greater chance to be causal. However, the incomplete penetrance or variable expression of a clinical phenotype can explain the presence of a pathogenic CNV in an unaffected (or sub-clinically affected) parent. As well as a “de novo” event is indicative, but not necessarily the cause of the disorder.

**Figure 6:**
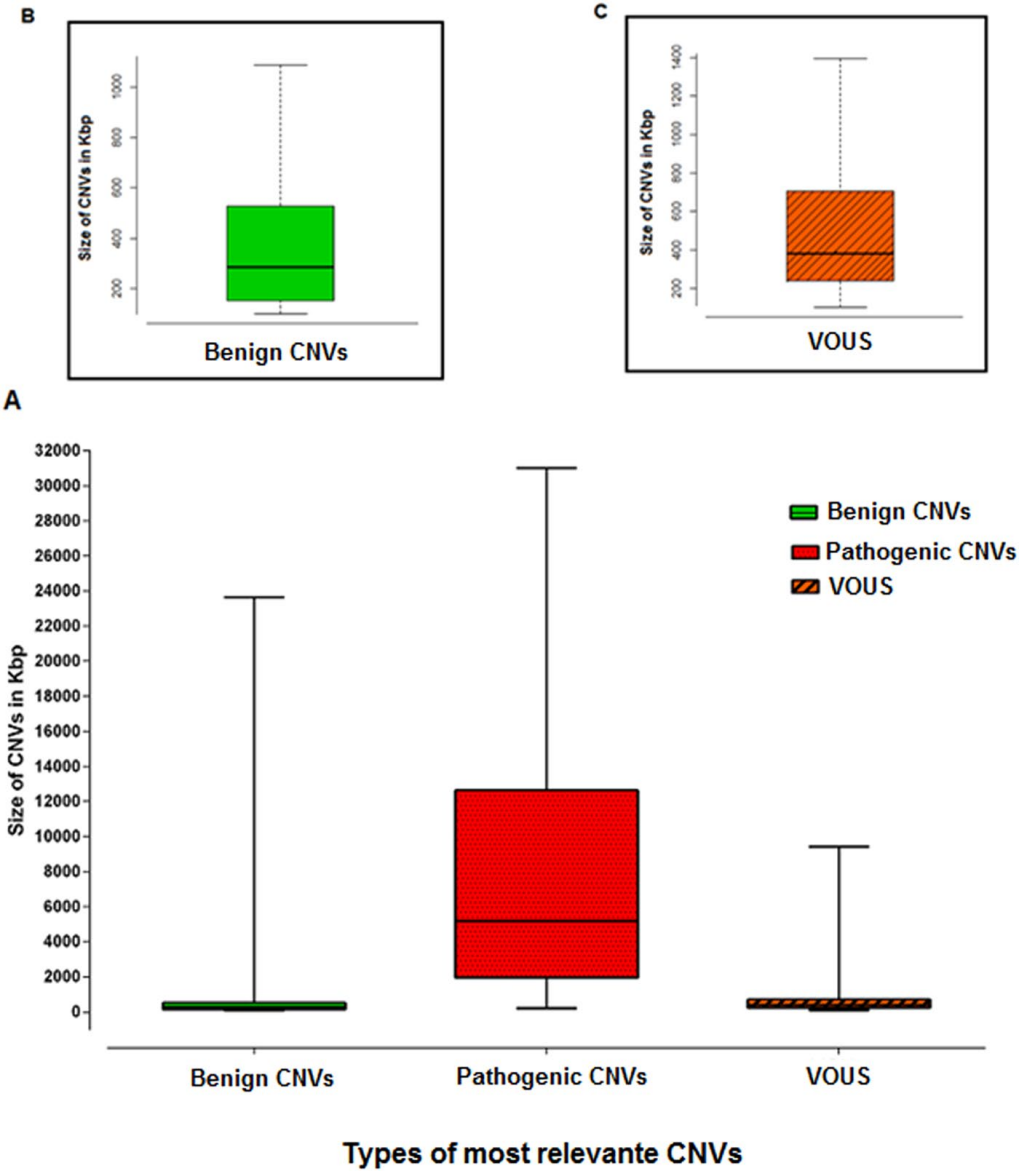
(**A**) CNV type by size variation. (**B**) Benign CNV size variation on a larger scale. (**C**) VOUS size variation on a larger scale.

In regard to their size, the pathogenic CNVs were typically very large (Fig. 6A), with a mean size of 7,770 kbp (median: 5,179 kbp), and contained multiple genes when compared with benign CNVs (mean: 483 kbp, median: 285 kbp, Fig. 6A,B) and VOUS (mean: 666 kbp, median: 382 kbp), as shown in Fig. 6A,C, in agreement to findings by others^25,48,49^. The variation inside each class is very large and some pathogenic CNVs are quite small whereas some benign CNVs can be very large when they are situated in gene-poor regions, like those close to centromeres. It is to expect that a VOUS is not typically very large because the more genes a CNV contains the higher chance of including known dosage-sensitive genes, regulatory regions or, in case of a deletion, to expose a recessive mutation which may be present in the remaining copy of the gene.

Based on the clinical data, obtained from the medical records, the most frequent phenotypes reported are also the main reasons of referral: DD, ID, congenital anomalies and/or dysmorphia, and ASD (Table 4). The same phenotypes are predominant in other CMA studies for the investigation of neurodevelopmental disorders^4,5,9,11,14–18^.

For instance, congenital anomalies, along with facial dysmorphisms, were reported in more than 58% of our cohort (Table 4). This frequency similar if the findings of 50% in a cohort of 78 affected with ND in the study of Qiao *et al*.^50^ and the 55% reported by Roselló *et al*.^5^ in their study with 246 patients with DD and ID, and probably represents a selection bias by the MDs for the referral for testing. Nevertheless, there was no statistical difference of diagnostic rate for patients with neurodevelopmental disorders without an obvious congenital anomaly or dysmorphia (data not shown).

Univariate analysis showed a significant association for the presence of pathogenic CNVs with dysmorphic facial features (*p*-value = < 0.0001, OR = 0.32) and ASD (*p*-value = 0.039). Congenital anomalies only showed a higher association with pathogenic CNVs in this cohort when broken down into more specific affected systems, where genitourinary anomalies had a higher correlation with the finding of a pathogenic CNV (*p*-value = 0.032). Furthermore, two secondary phenotypes, obesity (*p*-value = 0.006) and short stature (*p*-value = 0.032), were shown to be phenotypes associated to higher findings of pathogenic CNVs in patients with ND. However, this are incipient results, and should be avoided to be used for testing decisions. A clinical and standardized reassessment in all cases and a larger sample would be crucial to confirm this.

As already discussed by Quintela *et al*.^26^ the interpretation of genomic variations such as CNVs is an arduous task, especially in the challenging VOUS, when the genotype is suggestive of a genomic disorder characterized by incomplete penetrance and/or variable expressivity.

Regarding the negative diagnoses of the CMA (without CNVs or with only benign CNVs) in high resolution SNP CMA platforms like the ones used in this study, the homozygous regions can also be studied. Those results with very large LCSHs (long contiguous stretches of homozygosity) indicating possible uniparental disomy (UPD) or consanguinity should be reported to the accompanying MD for follow-up investigation of eventual imprinting syndromes or autosomal recessive mutations, through methylation or exome analysis. The relevance of LCSHs, which can be identified by most modern CMA platforms, is discussed elsewhere^51^.

## Conclusions

The diagnostic rate for CMA in this study was 18% and is within the literature (15–20%). CMA is an essential tool to decipher the sequences involved in structural karyotype abnormalities detected by classical chromosome analysis, as well as patients with abnormal CMA results should have their chromosomes analyzed - which can lead to unexpected surprises. For a correct diagnosis CMA and chromosome analysis should be used complementary. Parental chromosome analysis is essential for genetic counselling, particularly when the patient has terminal deletion/duplication or large CNVs. The main reasons for referral for CMA testing were DD/ID, dysmorphic facial features and ASD. Dysmorphic facial features and ASD (as main or secondary feature) and secondary phenotypes such as obesity, short stature, genitourinary anomalies are possible predictive phenotypes of a higher diagnostic answer by CMA.

Clinical interpretation of CNVs is still a challenge and depends in large part on information about their frequency in normal and affected populations, provided by cohort studies with significant samples.

## Methods

### Ethical aspects

The project was submitted and approved by the Research Ethics Committee of the Hospital Infantil Joana de Gusmão, the children´s hospital of Florianópolis-SC, Brazil, under the Nr 2,339,104, and respects the guidelines and criteria of the resolution Nr 466/12 of the Brazilian National Health Council. Patients or their parent and/or legal guardian (in cases where patient was under legal age), signed the Informed Consent Form. In cases in which it was not possible to contact the patient for any justifiable reason (loss of contact information, mainly) the data was used and a Justification of Absence of Consent approved by Research Ethics Committee and signed by the research team, ensuring the commitment to maintain confidentiality and privacy of the patients whose data and/or information was collected in the records.

### Sample

The sample refers to the reading files of CMA and available clinical data from 420 patients from the south of Brazil, mostly children, with neurodevelopmental disorders. The CMAs were requested by medical geneticists and neurologists for diagnostic purposes, mainly from the Joana de Gusmão Children’s Hospital, but also from the University Hospital Professor Polydoro Ernani de São Thiago and from private clinics in Florianópolis (State of Santa Catarina), throughout the years 2013 to 2016 and performed by the Laboratório Neurogene (Florianópolis, Santa Catarina, Brazil).

### Collection of clinical data

To correlate the phenotype to possible causal genes, the clinical description of the affected individuals was collected with their MDs through a questionnaire, seeking information about their clinical presentation, behavior, history of physical exams, as well as results of previous genetic and metabolic tests and prescription medication. No new appointments with the patients were made for this, and clinicians retrieved most data from their medical records.

### Genomic analysis

The platforms used were CYTOSCAN 750K (75%) and CYTOSCAN HD (25%) and the resulting files were analyzed using the CHROMOSOME ANALYSIS SUITE (ChAS) AFFYMETRIX software, based on the reference genome sequence of the University of California, Santa Cruz database (https-//genome.ucsc.edu/cgi-bin/hgGateway) using the human genome version of February 2009 (GRCh37/hg19). The filter criteria for CNVs were sizes >100 Kbp for deletions and >150 Kbp for duplications, both with at least 50 markers, according to ACMG recommendations^52^.

### CNVs interpretation and classification

To interpret CNVs, regarding their function, dosage effects (known haploinsufficiency or overexpression studies) and effects of mutations, the UCSC Genome Browser with integrated databases was widely used, mainly ClinVar (NCBI), DECIPHER (Database of Chromosomal Imbalance and Phenotype in Humans using Ensembles Resources), DGV (Database of Genomic Variants), OMIM (Online Mendelian Inheritance in Man), ISCA (International Standard Cytogenomic Array), dbGaP (Database of Genotypes and Phenotype), dbVAR (Database of Large Scale Genomic Variants), ECARUCA (European Cytogeneticists Association Register of Unbalanced Chromosome Aberrations), PUBMED (Public Medline), ClinGen (Clinical Genome Resource), MGI (Mouse Genome Informatics Database, from The Jackson Laboratory) and the private database CAGdb (Cytogenomics Array Group CNV Database).

The variants were classified into three types according to clinical interpretation as benign, variants of uncertain significance (VOUS), or pathogenic variants (causal), and the result in each case was assigned based on the CNV(s) of greatest clinical relevance detected in the genome of the patients.

Variables like location, type and size of each CNV, the CNV classification, number of CNVs detected for each patients, age, gender, clinical descriptions (phenotypes), previous genetic testing results (karyotype, fragile X, etc.), and other relevant known clinical data, were compiled (with coded identification) into simple Excel sheet for data handling with the R software (version 3.4.2, the R FOUNDATION FOR STATISTICAL COMPUTING) in order to understand the phenotypic frequency, the diagnostic rate of the study, the average age and the gender distribution in the cohort, the frequency of genomic changes in each chromosome, and the relation of the phenotype (or groups of clinical phenotypes) to the type of CNV to find if there are any indications which allow to recognize the patients with higher chance of carrying a pathogenic CNV - most suitable for submission to the CMA as a first-line test in the unfortunate setting of financial shortage.

### Ethics approval and consent to participate

The project was submitted and approved by the Research Ethics Committee of the Hospital Infantil Joana de Gusmão, the children’s hospital of Florianópolis-SC, Brazil, under the Nr 2,339,104, and respects the guidelines and criteria established by the resolution 466/12 of the Brazilian National Health Council. Patients or their caregivers signed the Informed Consent Form to participate in the study. In cases in which it was not possible to contact the patient for any justifiable reason (loss of contact information, mainly) the data was used and a Justification of Absence of Consent was signed by the research team, ensuring the commitment to maintain confidentiality and privacy of the patients whose data and/or information was collected in the records.

## Data Availability

The datasets used and/or analyzed during the current study can be requested from the corresponding author on reasonable request. However, since the patients or their caregivers signed an Informed Consent Form specifying that the data will be used only for the present study, their use for another study necessarily implies a new submission to the ethics committee of the Hospital Infantil Joana de Gusmão and depends on a new approval.
